# Risk Factors for Acute Kidney Injury Following Cardiac Surgery and Performance of Leicester Score in a Spanish Cohort

**DOI:** 10.3390/jcm11040904

**Published:** 2022-02-09

**Authors:** Alícia Molina Andújar, Alvaro Lucas, Victor Joaquin Escudero, Irene Rovira, Purificación Matute, Cristina Ibañez, Miquel Blasco, Elena Sandoval, Jesús Ruiz, Marina Chorda Sánchez, Gaston J. Piñeiro, Eduard Quintana, Esteban Poch

**Affiliations:** 1 Nephrology and Kidney Transplantation Department, Hospital Clínic, IDIBAPS, University of Barcelona, 08036 Barcelona, Spain; amolinaa@clinic.cat (A.M.A.); vjescudero@clinic.cat (V.J.E.); MIBLASCO@clinic.cat (M.B.); gjpineir@clinic.cat (G.J.P.); 2 Faculty of Medicine and Health Science, Medicine Campus, University of Barcelona, 08036 Barcelona, Spain; alvarolucasi@hotmail.com; 3 Anesthesiology Department, Hospital Clínic, University of Barcelona, 08036 Barcelona, Spain; irovira@clinic.cat (I.R.); pmatute@clinic.cat (P.M.); CRIBANEZ@clinic.cat (C.I.); 4 Cardiovascular Surgery Department, Hospital Clínic, University of Barcelona, 08036 Barcelona, Spain; esandova@clinic.cat (E.S.); jeruiz@clinic.cat (J.R.); equintan@clinic.cat (E.Q.); 5 Perfusion Department, Hospital Clinic, University of Barcelona, 08036 Barcelona, Spain; mchorda@clinic.cat

**Keywords:** acute kidney injury, cardiac surgery, Leicester score, prediction, risk factors, intensive care unit

## Abstract

The incidence of acute kidney injury following cardiac surgery (CSA-AKI) is up to 30%, and it places patients at an increased risk of death. The Leicester score (LS) is a new score that predicts CSA-AKI of any stage with better discrimination compared to previous scores. The aim of this study was to identify risk factors for CSA-AKI and to assess the performance of LS. A unicentric retrospective study of patients that required cardiac surgery with cardio-pulmonary bypass (CPB) in 2015 was performed. The inclusion criteria were patients over 18 years old who were operated on for cardiac surgery (valve substitution (VS), Coronary Artery Bypass Graft (CABG), or a combination of both procedures and requiring CPB). CSA-AKI was defined with the Kidney Disease Improving Global Outcomes (KDIGO) criteria. In the multivariate analysis, hypertension (odds ratio 1.883), estimated glomerular filtration rate (EGFR) <60 mL/min (2.365), and peripheral vascular disease (4.66) were associated with the outcome. Both discrimination and calibration were better when the LS was used compared to the Cleveland Clinic Score and Euroscore II, with an area under the curve (AUC) of 0.721. In conclusion, preoperative hypertension in patients with CKD with or without peripheral vasculopathy can identify patients who are at risk of CSA-AKI. The LS was proven to be a valid score that could be used to identify patients who are at risk and who could benefit from intervention studies.

## 1. Introduction

The incidence of acute kidney injury (AKI) following cardiac surgery is up to 30%, and between 2% and 5% of cases require renal replacement therapy (RRT) during the AKI episode. AKI places patients at a five-fold increased risk of death during admission, and when there is a need for RRT, it is associated with a 50% mortality [1].

Identifying pre-operative and intraoperative risk factors for cardiac surgery-associated AKI (CSA-AKI) is a vitally important issue for both clinical practice and research in order to detect patients who are at risk and who can be the focus for intervention. Potentially modifiable intraoperative risk factors remain of equal importance since their identification can lead to actions towards them. One of the most important factors is the inadequate balance between oxygen delivery (DO_2_) and oxygen consumption (VO_2_), and factors associated with this imbalance include hemodilution, low hemoglobin levels, and hypotension [2].

On the other hand, a number of predictive scores based on preoperative risk factors have been developed, such as the Cleveland Clinic Score (CCS), the most widely used and validated tool for not only RRT prediction, but also for the identification of severe AKI. It was first developed in 2012, but more recently, nadir intraoperative hematocrit has been added in order to improve the area under the curve (AUC), especially for patients without preexisting chronic kidney disease. The original score considers sex, presence of congestive heart failure, low left ventricular ejection fraction, preoperative use of intra-aortic balloon pump (IABP), chronic obstructive pulmonary disease (COPD), previous cardiac surgery, emergency surgery, type of surgery, and preoperative creatinine [3].

Another well-established predictive score is the European System for Cardiac Operative Risk Evaluation II (EuroSCORE II), a cardiac risk model for predicting mortality after cardiac surgery that was updated in 2012 from the previous EuroSCORE published in 1999 [4]. Even though it is not its aim, studies have shown an association between EuroSCORE and CSA-AKI [5], but again, it is better able to discriminate severe AKI.

Even though AKI that requires RRT is the scenario with the worst outcome, non-severe AKI, the most common form of AKI associated with cardiac surgery, has also been proven to be independently related to all-cause mortality [6]. Birnie et al. created the Leicester score (LS) to predict CSA-AKI of any stage in a British cohort, and it showed better discrimination ability compared to Euroscore II and the CCS [5]. The model included age, sex, BMI, smoking habits, dyspnea, diabetes, peripheral vascular disease, hypertension, preoperative hemoglobin, preoperative estimated glomerular filtration rate, time from catheterism to surgery, presence of triple vessel disease, ejection fraction, emergency surgery, and type of surgery. This scoring system was created in 2014, and since then, it has only been used to identify patients who are at risk in one German study, which was published in 2020 by Grieshaber et al., in which the prediction of all stages of AKI was not possible using the CCS but was possible when using the LS [7]. The variables that are included in the three mentioned scores are described in Table 1.

The aim of this study was to identify risk factors for CSA-AKI in a Spanish cohort and to assess the performance of the LS in the same cohort.

## 2. Material and Methods

We conducted a unicentric retrospective study of patients admitted to Hospital Clínic de Barcelona requiring cardiac surgery with cardio-pulmonary bypass (CPB) from January 2015 to December 2015. The inclusion criteria were patients over 18 years old who were operated on for cardiac surgery (valve substitution (VS), Coronary Artery Bypass Graft (CABG), or a combination of both procedures requiring CPB. All stages of chronic kidney disease were included. Patients who were already in chronic dialysis therapy, renal transplant recipients, or those who suffered an AKI just before the surgery were not included in the study. Additionally, emergent surgeries, patients with IABP use, patients who died during surgery, and patients with endocarditis were excluded. The Ethics Committee of our institution approved the study.

### 2.1. Data Colection and Definitions

Clinical, epidemiological, and laboratory variables were collected from the Electronic Health Records of our institution (SAP^®^). For every patient, data regarding their medical history, surgical characteristics, intraoperative variables, 24 h monitoring in the intensive care unit (ICU), and renal function evolution until discharge were collected. For patients requiring RRT, its duration was also recorded.

The LS, CCS, Euroscore II, and Charlson Index were calculated with the information from the pre-anesthesia visit and/or patient admission report.

The intraoperative variables that were recorded were aortic cross-clamp time (ischemia time) and CPB time, use of furosemide, use of vasoactive drugs, or need for transfusion. The post-operative variables included monitoring of the first 24 h in the ICU and whether the patient was consistent in terms of the use of vasoactive drugs, use of furosemide, and need for transfusion. Finally, seriated serum creatinine (sCr) and estimated glomerular filtration rate (eGFR) values were collected throughout the admission period.

### 2.2. Definitions

CSA-AKI was defined using the Kidney Disease Improving Global Outcomes (KDIGO) criteria: serum creatinine increase ≥0.3 mg/dl within 48 h or ≥1.5- to two-fold sCr increase from baseline within one week after surgery. Due to the nature of the study, the urinary output criteria were not included. Moderate AKI was defined as a sCr increase of 2.0–2.9 times from baseline. Severe AKI was defined as a sCrincrease of ≥3 times from baseline, an increase of 0.5 mg/dL if baseline sCr ≥ 4.0 mg/dl, or initiation of RRT [8]. Baseline creatinine was considered as the value obtained 24 h before surgery. AKI duration was counted since the AKI diagnosis until sCr returned to the baseline value with or without an increase of 0.3 mg/dL.

### 2.3. Statistics

The study variables are expressed as mean ± standard deviation (SD) if a normal distribution was observed or as the median and interquartile range (IQR)otherwise. Categorical variables were expressed as an absolute value (n) and relative (%) frequency. P values less than 0.05 were considered significant. Variables associated with the risk of CSA-AKI were assessed by logistic regression in univariate analysis, and those with statistical significance were included in the multivariate analysis, excluding score variables. To test the performance of the LS, we calculated the area under the receiver-operating characteristic curve (AUC) to assess the discrimination. We applied the Hosmer–Lemeshow goodness-of-fit test to assess its calibration. To compare ROC curves, we used DeLong’s test. A P-value above 0.05 indicates acceptable calibration. The statistical analysis was conducted using SPSS v.25 (SPSS Inc, Chicago, IL, USA).

## 3. Results

### 3.1. Baseline Characteristics

During the study period, 444 patients were included. Table 2 shows the baseline characteristics of the overall population. A total of 64.2% of the patients were male with an age ≥75 years old in 30.9% of the cases and a median age of 69 years. Hypertension was the most prevalent comorbidity (76.1%), with obesity being observed in 34% of patients and diabetes being observed in 35.3% of patients. As for peripheral vascular disease, it was only diagnosed in 9.5% of the patients. Median baseline creatinine was 0.9 mg/dL (IQR 0.73–1.06), and 19.4% of the patients had an eGFR <60 mL/min. The most common procedure was VS, and 10.6% of the patients had undergone previous cardiac surgery. Anemia (hemoglobin <120g/L) was present in 19.6% of the patients before cardiac surgery.

### 3.2. Surgical Characteristics and Post-Intervention Lengh of Stay

Surgical characteristics included a median CPB time of 91.5 (IQR 72–117) minutes and a median ischemia time of 65 (80–80.25) minutes. During the intervention, 115 patients (26%) required blood transfusion. As for vasoactive drugs, phenylephrine and or noradrenaline were used in 67% of the patients, dobutamine was used in 44.2%, and nitroglycerine/nitroprusside was used in 34.5%. Furosemide was used in 114 patients (25.7%), and ultrafiltration was used in 14 (3.15%).

As for the first 24h in the ICU, furosemide was used in 201 patients (45.9%). The most common vasoactive agents were vasodilators (nitroglicerine or nitroprusside), which were used in 42.3% of the patients, followed by dobutamine, which was used in 45.2% of the patients, followed by noradrenaline, which was administer to 35% of the patients.

The post-surgical length of stay was a median of 9 days (IQR 7–13), and 9 patients died during admission (2%).

### 3.3. AKI Characteristics

A total of 171 patients (38.5%) developed AKI during the first week after cardiac surgery. Its characteristics are described in Table 3. Of note, the most frequent form of AKI was mild AKI, which was present in 105 patients (61.4%). From all of the cases of mild AKI, almost 50% only met the “>0.3 mg/dL in 48h” criteria. Stage 3 AKI developed in 26 patients, 15 of whom required RRT. A total of 66.7% of the AKI cases met the criteria within the first 24 h, and the median duration until total recovery was 3 days. Two patients required dialysis at discharge. There were no differences in the baseline and discharge sCr and EGFR in all of the patients who were discharged without RRT (0.85 (0.68–1.04), 87 (64–91), respectively).

### 3.4. Preoperative and Intraoperative Risk Factors for CSA-AKI

A univariate analysis was performed to identify preoperative and intraoperative risk factors to assess the performance of the different risk scores (CCS, LS, and Euroscore II). Table 4.

Of the included preoperative risk factors, those who were associated with CSA–AKI were over 75 years of age (odds ratio (OR) 1.868 (1.24–2.815), *p* = 0.003), had hypertension (OR 2.172 (1.334–3.535), *p* = 0.002), peripheral vascular disease (OR 4.085(2.058–8.106), *p* < 0.001), anemia (OR 2.099 (1.307–3.372), *p* = 0.002), and higher sCr and lower EGFR (OR 6.778 (3.405–13.49) and OR 0.964(0.955–0.975), respectively, *p* <0.001). Lower eGFR remained significant when categorized for EGFR < 60 mL/min. Bypass surgery alone was the only variable associated with lower incidence of AKI (OR 0.642 (0.43–0.958), *p* = 0.03.

The three scores were significantly associated with CSA-AKI, with an OR of 1.058 (1.042–1.073) for the LS, 1.203 (1.103–1.306) for Euroscore II, and 1.188 (1.081–1.306) for the CCS. The Charlson index was also significantly associated with AKI, with an OR of 1.373 (1.226–1.537).

Of the included intraoperative risk factors, the need for blood transfusion was significantly associated with the outcome (OR 1.610 (1.047–2.477), *p* = 0.03), as was the use of noradrenenaline or phenilephrine (OR 1.543 (1.039–2.292), *p* = 0.032), while the use of vasodilators such as ntroglycerine or nitroprusside appeared to be protectors (OR 0.576 (0.379–0.873), *p* = 0.009). Longer CPB and ischemia times were also associated with the outcome (OR 1.007 (1.002–1.012), *p* = 0.005 and OR 1.012 (1.005–1.018) *p* < 0.001, respectively).

The statistically significant variables were introduced in a multivariate logistic regression analysis, where the variables that remained associated with the outcome were: hypertension (OR 1.883 (1.086–3.265), *p* = 0.024), EGFR < 60 mL/min (2.365 (1.375–4.070), *p* = 0.002), and peripheral vascular disease (4.66(2.134–10.177), *p* < 0.001). Ischemia time >70 min almost reached statistical significance, with a *p* value of 0.058. (Table 5).

### 3.5. Leicester Score Performance

**Figure 1:**
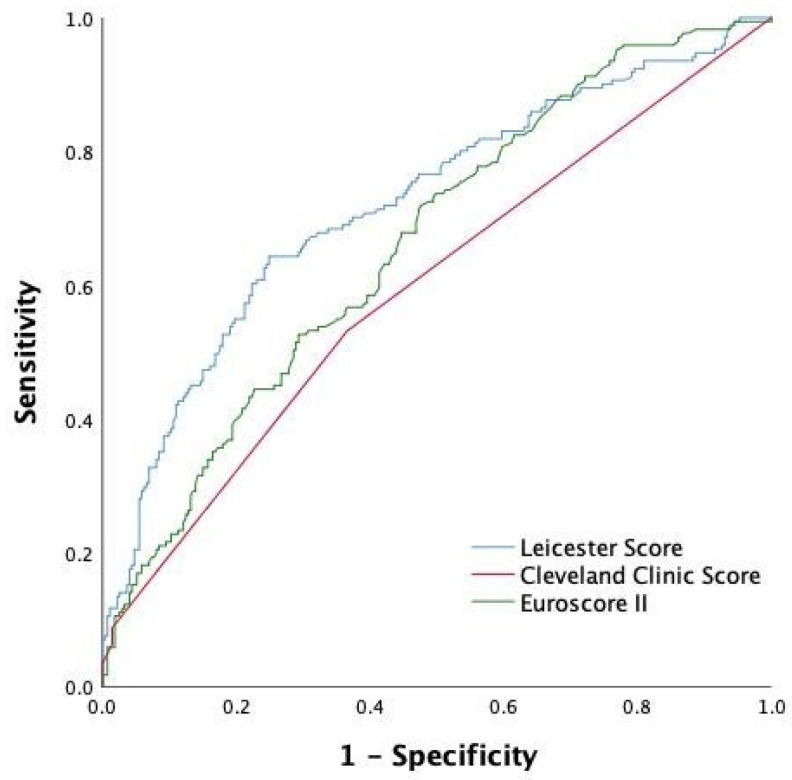
Receiver operating characteristic curve of each scoring system (Leicester score, Cleveland Clinic score, and Euroscore II).

The diagnostic utility of the Leicester score was compared with two previously published scores that have been widely validated: the CCS and Euroscore II. Using the original formula, we found an AUC of 0.721 (0.671–0.771) for any grade of CSA-AKI and a goodness-of-fit χ^2^ = 10.61 (*p* = 0.225) for the LS. Both discrimination and calibration were better compared to the CCS and Euroscore II (Table 6, Figure 1), and the difference between curves was statistically significant (Table 7). Compared to the original validation cohort, LS had similar accuracy (AUC 0.73).

## 4. Discussion

In this retrospective unicentric study, we evaluated risk factors for CSA-AKI and assessed the performance of the LS in a Spanish cohort. Preoperative hypertension, chronic kidney disease (CKD), peripheral vascular disease, and longer ischemia time were the variables that remained associated with the outcome. The LS showed the best discrimination and calibration compared to classic scores.

Our results do not differ from classical studies. In 2016, Yi Q et al. conducted a meta-analysis of the studies that assessed the risk factors for CSA-AKI (CS and/or CABP surgeries with CPB). The authors identified hypertension, preoperative sCr level, peripheral vascular disease, respiratory system disease, diabetes, cerebrovascular disease, low cardiac output, New York Heart Association (NYHA) classification class III/IV, emergency surgery, infection, re-intervention, and use of IABP as preoperative risk factors for CSA-AKI [9].

Since the presence of CKD seems to be one of the most important risk factors in the different cohorts, specific studies have also been performed in order to identify risk factors for patients with impaired kidney function. In 2020, Fu HS et al. described the risk factors for CSA-AKI in patients with eGFR < 30 mL/min, showing that only a high preoperative serum creatinine and decreased CPB target temperature were significant risk factors for postoperative AKI in their multivariate analysis [10]. On the other hand, other studies showed that baseline sCr seem to be associated with CSA-AKI, only in patients with eGFR < 60 mL/min [11].

CPB is a major intraoperative factor contributing to CSA-AKI. The pathophysiology of kidney injury during CPB is complex and multifactorial. There are four main pathways that lead to AKI during CPB: oxygen supply/demand mismatch, the activation of an inflammatory response, haemolysis, and lipid microemboli [12,13,14,15]. All of these mechanisms explain why longer CPB and ischemia times are classically associated with AKI [16].

Hemodynamics remain of vital importance in cardiac surgery with CPB. Renal oxygenation is determined by an equilibrium between oxygen delivery (RDO_2_) and oxygen consumption (RVO_2_). Cardiac output (CO) and oxygen arterial content (CaO_2_) are the two main determinants of RDO_2_. Therefore, the influence of low hematocrit [17] and low mean arterial pressure on CSA-AKI can be pathophysiologically explained. Nevertheless, there is inconclusive evidence on the relationship between the MAP targeted during CPB and the incidence of CSA-AKI [18]. On the other hand, studies have shown that a greater difference in intraoperative MAP relative to preoperative MAP can be a risk factor. In a prospective study of 157 patients, Kenji et al. found that a drop in MAP of >26  mmHg was independently associated with the development of AKI following cardiac surgery [19]. This could explain why preoperative hypertension was also a risk factor for CSA-AKI in our cohort and could be the focus of intervention. As for peripheral vascular disease, it is not only a known risk factor for AKI but for other major complications, especially in patients undergoing CABG [20].

There is a need to identify patients who are at risk for CSA-AKI in order to include them in clinical trials. Since mild AKI has also been proven to be an independent risk factor for mortality, scores that assess the risk of AKI of any stage in combination with biomarkers of early renal damage should be the focus when designing intervention studies.

In this regard, results from the PrevAKI trial were published in 2017. In that trial, a KDIGO guidelines based strategy was performed in high-risk patients undergoing cardiac surgery, who were defined as having a urinary Tissue inhibitor of metalloproteinases-2 (TIMP-2) x Insulin-like growth factor-binding protein 7 (IGFBP7) > 0.3. AKI was significantly reduced with this intervention compared to in the controls [21]. Biomarkers were used to identify patients who were at a high risk of clinical AKI in order to use preventive strategies for the first time.

As for the scores, the CCS was validated for the prediction of postoperative AKI requiring RRT, although it has been used in other studies to predict all stages of AKI [3]. In contrast, the LS was validated for the prediction of all stages of AKI, as defined by KDIGO [5]. Hence, the last one appears to be the best method for the identification of a wider spectrum of patients.

The LS was first published in 2014. The model was developed using the Bristol and Birmingham center datasets, and it was externally validated using data from Wolverhampton center, which is also in United Kingdom. Diagnostic utility was compared to existing scores. The risk prediction score for any AKI stage (AUC = 0.74 (0.72, 0.76)) demonstrated better discrimination compared to the Euroscore and the CCS, and better calibration was also observed.

It was not until 2020 that this score was first used in an external study conducted by Grieshaber P et al. in a German center [7]. In the study, the authors tried to identify patients who were at a high risk for CSA-AKI by combining clinical risk stratification with either the CCS and LS and the early postoperative quantification of urinary biomarkers for AKI using (TIMP-2)·(IGFBP7). Although the LS was predictive for all stages of AKI, the CCS was only predictive for stage 2 or 3 AKI. In this study, its AUC (0.601) was poor, so the LS performed markedly worse compared to in the validation cohort (AUC 0.73). Urinary (TIMP-2)_(IGFBP-7) quantification 4 h postoperatively did not add value to the predictive value of the clinical score. By contrast, in our study, the AUC (0.72) was similar to the validation cohort in the original study. This could be due to the higher EuroSCORE values in the German study (2.5 for no-AKI and 2.8, 6.0 and 8.8 for stages 1–3, respectively) compared to in our cohort. Even though the median values from the validation cohort are not available, they could be closer to our cohort. Based on the first external study, the performance of the LS could be considered weak, but our good results make the LS a promising scoring system that can be used by clinicians in future intervention studies in order to identify patients who are at risk for AKI.

Our study has some limitations. First, patients who were in a critical state (requiring emergency surgery or the use of IABP) or who had endocarditis were excluded. These patients were not excluded in the study where the LS was first validated, which makes our cohort not completely comparable. Critical patients are not the focus for clinical trials, so the good performance of the LS in our cohort could serve as the basis for designing intervention studies in patients wo are susceptible to developing AKI. This is a retrospective study that provides information about LS performance in a large cohort of patients, but prospective studies are needed in order to confirm and externally validate the LS.

In conclusion, based on our results, preoperative hypertension in patients with CKD with or without peripheral vasculopathy could be a focus for intervention in future studies. The LS has proved to be a valid scoring system that could be used to identify patients who are at risk who could benefit from future intervention studies.

## Figures and Tables

**Table 1:** Variables included in the EuroSCORE II, Cleveland Clinic Score, and Leicester score.

Euroscore II	Cleveland Clinic Score	Leicester Score
Age	---	Age
Gender		
Preoperative renal function (Cockroft–Gault formula, ml/min): >85, 50–85, <50, dialysis treatment	Preoperative renal function (creatinine, mg/dL): <1.2 mg/dL, 1.2–2.1, ≥2.1	Renal function (Cockroft–Gault formula, ml/min): >90–60–89, 30–59, <30
Poor mobility	---	---
Chronic lung disease	COPD requiring treatment	---
Previous cardiac surgery	Previous cardiac surgery	---
Active endocarditis	---	---
Critical preoperative state	Preoperative use of IABP	---
Diabetes mellitus on insulin therapy	Diabetes mellitus on insulin therapy	Diabetes mellitus
NYHA class (I–IV)	Heart failure	NYHA class (I–IV)
Class IV angina ^a^	---	---
Left ventricular function (%): >50, 31–50, 21–30, <21	Left ventricular function <35%	Left ventricular function (%): ≥50, 40–49, <40
Recent miocardial infarction (90 days)	---	---
Pulmonary hypertension: ystolic arterial pressure 31–55 mmHg, >55	---	---
Urgency; (elective, urgent, emergency, salvage)	Emergency surgery	Urgency (elective, urgent, emergency)
Type of surgery: isolated CABG, non-CABG, 2 procedures, 3 procedures	Type of surgery: CABG, valve, CABG + valve, other	Type of surgery: CABG, single valve, CABG + valve, other/multiple
Surgery on thoracic aorta	---	---
---	---	Body mass index (kg/m^2^): <20, 20–24, 25–29, 30–34, >34
---	---	Smoking habit: never, ex-smoker, current
---	---	Hypertension
---	---	Peripheral vascular disease
---	---	Preoperative hemoglobin (g/dL) (<10, 10–11.9, ≥12)
---	---	Triple vessel disease
---	---	Time from catheterism to surgery

COPD: chronic obstructive pulmonary disease, CABG: coronary artery bypass grafting; IABP: intra-aortic balloon pump; NYHA: New York Heart Association. (a) Canadian Cardiovascular Society criteria.

**Table 2:** Baseline characteristics.

n = 444	n (%)/Median (IQR)/Mean+/−SD
Sex (%man)	285 (64.2)
Age (years) ≥75 years	69 (61–76) 137 (30.9)
History of smoking habit	213 (49)
Diabetes Diabetes with insulin therapy	157 (35.36) 42 (9.45)
Hypertension	338 (76.1)
BMI (kg/m^2^) BMI ≥ 30	28.33+/−4.47 136 (34.1)
Anemia Hemoglobin (g/L) Hematocrit (%)	87 (19.6) 134 (123–143) 39 (36–42)
Peripheral vascular disease	42 (9.5)
Low ejection fraction (<40%)	45 (10.13)
Creatinine (mg/dL) EGFR(ml/min) EGFR<60 mL/min CKD EIII CKD EIV	0.9 (0.73–1.06) 83.3 (65–91) 86 (19,37) 75 (87.2) 11 (12.8)
Previous cardiac surgery	47 (10.6)
Procedure	Valve surgery: 199 (44.8), CABG: 171 (38.5) Valve + CABG: 74 (16.7)
Charlson index	4 (3–5)
Euroscore II	1.77 (1.08–3.02)
Cleveland Clinic Score	0.4 (0.4–1.8)
Leicester Score	18.45 (11.12–30.94)

BMI: body mass index; EGFR: estimated glomerular filtration rate; CKD E III: chronic kidney disease stage III; CKD EIV: Chronic kidney disease stage IV; IQR: interquartile range; SD: standard deviation; CABG: coronary artery bypass grafting.

**Table 3:** Summary of acute kidney injury characteristics.

**AKI Stages**	**Days between Surgery and AKI Start**
AKI stage 1: 105 (61,4%)→49 met only the “>0.3 mg/dL in 48h” criteria (46.2%) AKI stage 2: 40 (23.4%) AKI stage 3: 26 (15,2%)→15 with dialysis requirement (57.7%)	Median time from surgery to AKI (1 (1–2) First 24 h: 114 (66.7%) 48 h: 36 (22.8%) 72 h: 10 (5.85%) >72 h: 11(6.4%)
**AKI duration**	**Dialysis technique**
Median duration time (days): 3 (1–6) 24 h: 50 patients (29.24%) 48 h: 26 patients (15.2%) 72 h: 23patients (13.45%) >72 h: 72 patients(42.1%)	Intermittent hemodialysis: 6 patients CRRT: 5 patients Both: 4 patients * Median intermittent hemodialysis sessions: 2 (IQR 1–4) Median CRRT treatment (days): 3 (1–4)

AKI: acute kidney injury; CRRT: continuous renal replacement therapy.

**Table 4:** Risk factors for cardiac surgery-associated acute kidney injury: univariate analysis.

	Total (n = 444)	No AKI (n = 273, 61.5%)	AKI (n = 171, 38.5%)	OR (IQR)	*p* Value
PREOPERATIVE					
Age ≥ 75 (years)	137 (30.9)	70 (25.6)	67 (39.2)	1.868 (1.24–2.815)	0.003
Sex (%Male)	285 (64.2)	168 (61.5)	117 (68.4)	1.354 (0.904–2.029)	0.142
BMI > 30	136 (34.1)	76 (31.3)	60 (35.1)	1.373 (0.901–2.093)	0.140
Ever smoked	213 (49)	130 (48.7)	83 (48.5)	1.029 (0.699–1.514)	0.884
Diabetes	157 (35.36)	89 (32.6)	67 (39.4)	1.365 (0.917–2.031)	0.125
Hypertension	338 (76.1)	194 (71.1)	144 (84.2)	2.172 (1.334–3.535)	0.002
Peripheral vascular disease	42 (9.5)	13 (4.8)	29 (16.9)	4.085 (2.058–8.106)	<0.001
EF < 40%	45 (10.13)	22 (8.1)	23 (13.4)	1.773 (0.9455–3.292)	0.070
Anemia	87 (19.6)	41 (15)	46 (26.9)	2.099 (1.307–3.372)	0.002
Creatinine (mg/dL)	0.9 (0.73–1.06)	0.86 (0.7–1)	0.99 (0.79–1.25)	6.778 (3.405–13.49)	<0.001
eGFR (ml/min)	83.3 (65–91)	85 (71–91)	72 (53–86)	0.964 (0.955–0.975)	<0.001
EGFR < 60 mL/min	86 (19.37)	32 (11.7)	54 (31.6)	3.571 (2.190–5.822)	<0.001
Only CABG	171 (38.5)	116 (42.5)	55 (32.2)	0.642 (0.43–0.958)	0.03
Leicester score	18.45 (11.12–30.94)	15.17 (9.2–22.45)	26.81 (16.4–41.42)	1.058 (1.042–1.073)	<0.001
Euroscore II	1.77 (1.08–3.02)	1.42 (0.95–2.61)	2.34 (1.34–3.89)	1.203 (1.103–1.306)	<0.001
Cleveland Clinic Score	0.4 (0.4–1.8)	0.4 (0.4–1.8)	1.8 (0,4–1.8)	1.188 (1.081–1.306)	<0.001
Charlson Index	4 (3–5)	3 (2–5)	4 (3–6)	1.373 (1.226–1.537)	<0.001
INTRAOPERATIVE					
Blood transfusion	115 (26)	61 (22.4)	54 (31.6)	1.610 (1.047–2.477)	0.030
Vasodilator agents	153 (34.5)	107 (39.2)	46 (26.9)	0.576 (0.379–0.873)	0.009
Dobutamine	198 (45.2)	118 (43.2)	78 (45.6)	1.114 (0.758–1.637)	0.584
Furosemide use	114 (25.7)	62 (22.7)	52 (30.4)	1.5 (0.974–2.310)	0.066
Vasoconstrictor agents	298 (67.12)	150 (54.9)	148 (86.5)	1.543 (1.039–2.292)	0.032
CPB time (min)	91.5 (72–117)	88 (71–110)	100 (74–127)	1.007 (1.002–1.012)	0.005
CPB time > 90 min	226 (51.4)	124 (45.8)	102 (59.6)	1.805 (1.222–2.666)	0.003
Ischemia time	65 (50–80.25)	60 (48–80)	75 (54–92)	1.012 (1.005–1.018)	<0.001
Ischemia time > 70 min	179 (41.2)	91 (33.8)	88 (51.5)	2.235 (1.504–3.324)	<0.001

OR: odds ratio; IQR: interquartile range; AKI: acute kidney injury; BMI: body mass index; EF: ejection fraction; EGFR: estimated glomerular filtration rate; CABG: coronary artery bypass grafting; CPB: cardiopulmonary bypass.

**Table 5:** Multivariate analysis of risk factors associated with cardiac surgery-associated acute kidney injury.

Variable	OR (CI)	*p*-Value
Age ≥ 75 years	1.483 (0.928–2.371)	0.099
Hypertension	1.883 (1.086–3.265)	0.024
EGFR < 60mL/min	2.365 (1.375–4.070)	0.002
Anemia	1.642 (0.918–2.939)	0.095
Bypass	0.838 (0,503–1.397)	0.499
Peripheral vascular disease	4.66 (2.134–10.177)	<0.001
Blood transfusion	0.87 (0.509–1.487)	0.608
Vasopressors agents	1.261 (0.784–2.027)	0.34
Vasodilators agents	0.694 (0.412–1.168)	0.169
CPB time >90 min	1.019 (0.553–1.879)	0.951
Isquemia time >70 min	1.844 (0.979–3.473)	0.058

OR: odds ratio; CI: confidence interval; EGFR: estimated glomerular filtration rate; CPB: cardiopulmonary bypass.

**Table 6:** Discrimination (area under ROC curves) for the different scores and calibrations (Hosmer–Lemshow tests).

	Discrimination		Calibration
	AUC (95% CI)	*p* Value	Chi Square *p* Value ^a^
Leicester score	0.721 (0.671–0.771)	<0.001	10.1 0.225
Cleveland Clinic Score	0.595 (0.54–0.65)	0.001	2.631 0.105
Euroscore	0.662 (0.611–0.713)	<0.001	11.48 0.176

^a^ Higher values indicate better calibration. ROC: receiver operating characteristic; AUC: area under the curve; CI: confidence interval.

**Table 7:** Comparison between ROC curves by DeLong’s test.

	CCS-Euroscore II	CCS-LS	Euroscore II-LS
Difference between areas	0.067	0.126	0.059
Stadard Error (CI)	0.025 (0.017–0.112)	0.030 (0.067–0.185)	0.027 (0.006–0.112)
Z statistic	2.633	4.203	2.199
*p*-Value	0.009	<0.001	0.028

ROC: receiver operating characteristic CCS: Cleveland Clinic Score; LS: Leicester Score; CI: confidence interval.

## Data Availability

The data that support the findings of this study are available on request from the corresponding author (E.P).
